# How does open innovation lead competitive advantage? A dynamic capability view perspective

**DOI:** 10.1371/journal.pone.0223405

**Published:** 2019-11-20

**Authors:** Kibaek Lee, Jaeheung Yoo

**Affiliations:** 1 Department of Research Planning, Korea Research Institute of Chemical Technology (KRICT), Daejeon, Republic of Korea; 2 Industry and Institution Research Group, Software Policy & Research Institute (SPRI), Seongnam, Republic of Korea; Shandong University of Science and Technology, CHINA

## Abstract

The relationship between open innovation and company’s competitive advantage, and organisational capabilities required remains to be explained. This study was conducted to answer the following questions. Does open innovation create organisation's competitive advantage? What types of capabilities are needed in the process of open innovation reaching competitive advantage, and what kind of relationship do they have? This study extends the scope of theoretical discussion about open innovation from the point of dynamic capability view. The results confirmed the statistical significance of the path linking open innovation to competitive advantage through product innovation. From the viewpoint of capabilities, transforming capability plays a role of significant prerequisite of sensing capability and seizing capability, having a direct or indirect significant effect on product innovation performance and competitive advantage sequentially. The results suggest that the linkages between the needed capabilities of organisation must be considered for performing open innovation to secure competitive advantage.

## Introduction

Over the past 10 years open innovation has attracted much attention from the industrial and academic communities as a method to secure the competitive advantage of organisations in the rapidly changing environment. Open innovation is a method for a company to achieve innovation based on the collaboration with a variety of knowledge sources that exist outside of it. The central idea behind open innovation is the creation and commercialization of innovative products that allow a company overcome its internal limitations and respond quickly to external changes by taking and absorbing external-origin knowledge [1]

The positive effect of open innovation on the achievement of product innovation is accepted in principle [2]. The process that open innovation leads to the competitive advantage of a company, however, has yet to be explained [3].

Since open innovation is also one of strategies adopted by enterprises to ensure continuous survival, the ultimate goal is to possess their sustainable competitive advantage. Securing competitive advantage for a company starts with identifying where the competitive advantage of its capability lies and how it can manage them [4]. From a perspective of open innovation, the competitiveness of an enterprise is determined by the difference in the ability or capability to utilize these resources in response to the rapidly changing environment as well as the size of resources it possesses [5].

Given that open innovation of a company requires the ability to consolidate, structure and reconstitute its resources, it can be explained in the same context as the dynamic capability view. The application of the viewpoint to look at organisational capability can draw many implications with respect to the establishment and management of open innovation strategies.

Unfortunately, previous researches on open innovation failed to understand the organisational capability that can lead to its success, indicating the low level of understanding what organisational capabilities are required for a company to secure competitive advantage through open innovation and how a company can accomplish the final outcomes with these capabilities.

This study aims at providing theoretical and practical implications on capabilities that are necessary for performing successful open innovation by establishing a comprehensive theoretical model incorporating a number of innovative activities performed by companies based on the interpretation of open innovation from the perspective of dynamic capability view.

## Literature review

### Competitive advantage (CA) and dynamic capability view (DCV)

Competitive advantage refers to the ability gained through attributes and resources to perform at a higher level than others in the same industry or market [4].

Many theories on the resources of corporate competitive advantage can be roughly divided into three categories: Industrial Organisation View (IOV) that intends to have a competitive advantage through positioning within the industrial competitive structure, Resource Based View (RBV) that seeks the source of success through the internal resources and core capability of a company and Dynamic Capability View (DCV) that can be explained as the ability to consolidate, structure and reconstitute capabilities exist inside and outside a company for the purpose of adapting to environmental changes.

Nowadays, companies have more difficulties in maintaining competitive advantage; even a company that has been successful in terms of technological lead is prone to lose its market dominance due to failing in new investment, the inertia of existing organisations or failed resource allocation [6]. In addition, due to the nature of resources to lock in the environment, it is difficult to convert resources into a suitable capability in reaction to the rapid changes in environment [7].

Increasing number of studies are paying more attention to DCV because it can better explain the competitive edges and business performance of companies through dynamic processes that create, consolidate, integrate, build and reconfigure internal and external competences to address rapidly changing environments [8].

### Open innovation (OPI)

One of the main goals of open innovation is to ensure a company’s swift response to competitive environment by collecting information of rapidly changing market and technologies [9]. Warning the advent of disruptive innovation led by some of low-function and low-cost technologies causing a possible threat to leading companies, Christensen (1997) puts stress on the importance of ability to sense these technologies [6].

Another goal of open innovation is to acquire knowledge for production innovation [10]. Traditionally, product innovation is based on the R&D capability that depends on the resources inside the company. However, it has become possible to accomplish product innovation more effectively by acquiring diverse and in-depth knowledge through external collaborations [11]. Considering the trend of the contemporary business environment where products have a short life cycle and the efficiency of closed-in-house R&D is decreasing [1], the ability to acquire and use necessary knowledge and skills in a cost-effective manner from the outside has become increasingly important.

Most previous studies focused on the relationship between open innovation and product innovation [2]. For example, Kang and Kang (2014) analysed the relationship between different knowledge sources and product innovation [10], and Hwang and Lee (2010) focused on the analysis of the effect of breadth or depth of external product knowledge on product innovation [12]. In addition, Tsai (2009) placed absorptive capacity between collaboration networks and product innovation performance as a moderator for the analysis [13].

In order to fully absorb the benefits of open innovation, it will be important for the organisation to have the capabilities it needs. However, previous researches on open innovation have not addressed the organisation’s ability that can lead a successful innovation [14]. Thus, there is a lack of understanding about required capabilities and the process to reach the final results while attempting to secure competitive advantage of company through open innovation.

### The relationship between OPI and DCV

Chesbrough (2003) emphasizes that open innovation should be accomplished to survive changes in the global knowledge landscape [1]. A company can acquire knowledge that exists outside the company and thereby improve the efficiency of innovation and risk management. It is also explained that technology and knowledge must be thoroughly reorganized in order to prevent the failure of open innovation, the relocation of resources helps firms to integrate internal and external knowledge [15].

Dynamic capability is composed of the combined capabilities and successive steps of specific organisational processes to implement strategies for creating new values using internal and external resources in the rapidly changing environment [8]. This study adopted the dynamic capability as the primary capability for open innovation with following reasons.

Dynamic capability is an organisation's ability to recognize potential technology changes and to adapt to changes through innovation in order to prepare for a changing business environment. The ability allows firms to identify opportunities and threats, explore knowledge and skills, and to proactively detect new market opportunities. Not only does it increase the ability to recognize potential technological changes, but it also increases the ability to adapt to changes by inducing innovation [16]. This is a fundamental capability required for open innovation.

The knowledge gained from the outside has a major effect on the competence of companies that develop innovative products. It is important, however, for companies to establish a process through which they can access, converge and share useful knowledge and skills both inside and outside the organisation because their technological innovation cannot be guaranteed by technological cooperation alone [14]. Furthermore, the process of choosing a business model and value-creation mechanism to develop new products and developing and allocating necessary resources, are essential to the success of open innovation [1].

Another element essential for open innovation is the capability that transforms the knowledge gained from the outside into the new knowledge by fusing it with the existing knowledge, and that enhances the evolutionary fitness to the environment utilizing exiting resources as new resources to cope with environmental changes [17].

Companies expect to benefit from the market by investing costs of open innovation and using the knowledge gained from collaboration for product innovation. From the perspective of dynamic capability view, open innovation success can be achieved from the organic combination of three types of capability; collection of information about rapidly changing market/technology to swiftly response to competitive environment (sensing capability), acquiring of knowledge for product innovation and performing of product innovation (seizing capability) and rearranging of resources to perform these activities effectively (transforming capability).

**Fig 1:**
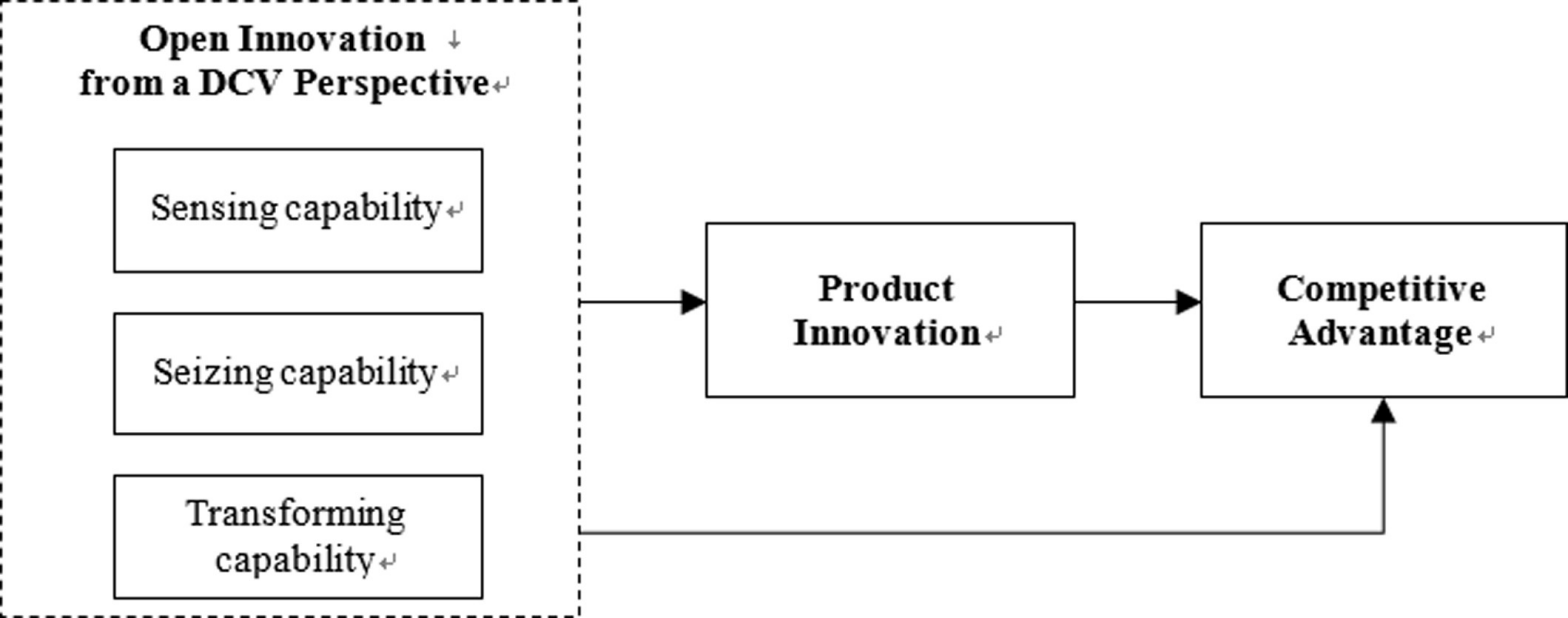
Conceptual framework of the study.

As mentioned earlier, previous studies about open innovation were not based on understanding from the viewpoint of organisational capacity. In this study, however, we examined the capabilities needed for open innovation from dynamic capability view perspective, and performed the measurement of variables, modelling and analysis. According to Teece (2007), dynamic capability is divided into three particular capabilities: sensing, seizing, and transforming capability [18]. The conceptual framework assumed in this study is illustrated in Fig 1.

## Hypotheses development and research model

### Dynamic capability (DC)

#### Sensing capability

Sensing capability is an ability needed for a company to identify opportunities for product innovation. If an organisation does not have a mechanism to recognize changes in the dynamic environment, the survival of the organisation may not be sustained. The scope of this cognitive activity should cover potential demand, the structural evolution of the industry and the market and the responses of both suppliers and competitors as well as the verification of in-house research activities, customer needs and technologies [18].

Companies are performing external knowledge sourcing as a part of sensing activities to respond to the changing environment [1]. They collect rapidly changing market and technology information and expect to acquire knowledge and skills for product innovation [10]. Product development has been based on R&D capability that depends on company’s internal human and physical resources. However, more effective production innovation activities have become possible by acquiring diverse and in-depth knowledge through external collaborations with various entities ranging from consumers to suppliers, research institutions, universities, and even competitors [11].

As such, external sensing capability provides opportunities to secure innovation through the collaboration with external partners. It is assumed, therefore, that sensing capability positively affects seizing capability and product innovation performance.

H1. Sensing capability positively affects seizing capability.
H2. Sensing capability positively affects product innovation performance.

#### Seizing capability

Seizing capability refers to an ability to seize sensed opportunities, select a business model to develop a new product, develop/allocate necessary resources, and commercialize the product [19]. Essential elements for seizing capability include market-oriented product development strategies, vertical integration strategies on the supply chain, clear strategic goals and leadership of managers and timely decision making [20].

A company with a strong seizing capability can select the most suitable opportunity at the right moment and create innovative results using various opportunities and knowledge identified through the sensing capability.

H3. Seizing capability positively affects product innovation performance.

#### Transforming capability

A company needs to transform resources that are the source of its competitiveness in order to respond to environmental changes. Transforming capability is the ability to integrate, reconstruct, renovate, create, and in some cases, dispose of existing resources for product innovation [18].

Becker and Dietz (2004) argue that the capability to coordinate, manage and control activities with various organisations is essential for successful external knowledge sourcing and innovation activities [21]. This means that the roles and relationships of the members, the procedures and structures of tasks should be reset. For example, P&G, IBM, Apple and GE have established organisations dedicated to collaborate with outside organisations. In addition, companies have built a knowledge management system to improve the efficiency of problem solving by allowing various internal and external-origin information to be exchanged and providing necessary information in a timely manner [22].

Such activities are required because it is difficult to effectively acquire and utilize external-origin knowledge without transforming capability for collaboration with the outside [21]. Therefore, transforming capability is a prerequisite to ensure that detection of internal and external changes and innovation activities are performed well, and an important innovation activity of companies to be carried out prior to strategic determination of sensing and seizing activities.

H4. Transforming capability will positively affect sensing capability.
H5. Transforming capability will positively affect seizing capability.

A general corporate innovation activity is to perform product innovation through internal R&D capability development in order to effectively acquire and utilize knowledge from inside and outside sources [11]. This internal R&D capability can be improved by strengthening transforming capability such as talent management, knowledge management, and creative management activity for innovation [23]. It is because the ability to integrate and coordinate external-origin technologies in relation to the internal technology when introducing those external technologies into the organisation, and the reorganisation of technology and knowledge contributes to the creation of innovative results by facilitating the integration of internal and external technologies [15].

Many companies are enhancing their product innovation through constant reinforcement of transforming capability. In order to improve the efficiency of innovation activities, they adopt measures to change their organisational structures such as organizing a task force team or placing human resources in the right positions as required [24]. They also identify best practices and spread them through company-wide education and training and enhance the efficiency of internal communication and work processes by introducing an information system for the purpose of acquiring more effective results of product innovation such as new product development and existing product enhancement.

H6. Transforming capability positively affects product innovation performance.

### Competitive advantage (CA)

#### Dynamic capability and competitive advantage

The achievement and maintenance of a company's high performance can be secured through recognizing and responding to opportunities from changes in the industrial environment [25]. Dynamic capability allows a company to find and utilize new resources that can be a source of competitive advantage, and resource reallocation and convergence, furthermore, play a critical role for it to achieve competitive advantage by enhancing responsive power [20]. In addition, a company’s capability to control knowledge is one of the key factors in achieving sustainable competitive advantage [22].

There are some of the previous studies on the relationship between DC and CA. Li and Liu (2014) performed an empirical study to analyse relationship between environmental dynamics, dynamic capability and competitive advantage, and explained the positive effects of company’s dynamic capability on its competitive advantage [26]. On the other hand, Wu (2010) divided the dynamic capabilities of companies into integration ability, learning ability and reconfiguration ability, and provided analysis results of the effects on competitive advantage indicating the positive effects of integration and reconfiguration abilities on competitive advantage [27]. Wu and Wang (2007) also argue that DC has a positive impact on competitive advantage [28]. Based on the above-mentioned research results, dynamic capability is expected to have a positive effect on the competitive advantage of the firm. Therefore, this study set the following hypotheses for the relationship between three elements of dynamic capability and competitive advantage.

H7. Sensing capability positively affects competitive advantage.
H8. Seizing capability positively affects competitive advantage.
H9. Transforming capability positively affects competitive advantage.

#### Product innovation performance and competitive advantage

Product innovation activities pursue the common goal of continuous survival and growth of companies, and whether or not a company can offer a superior value to competitors affects the purchase intention and behaviour of target customers. And the result is competitive advantage [29].

Product innovation refers to the development and release of goods or services based on customers’ needs or market demands. Product innovation provides differentiated competitiveness in terms of quality and function, which offers incentives for customers to choose. This allows companies to win competition, secure a market-leading position, and create market performance by attracting new customers [30].

As presented above, previous studies consider the innovation result of a company as a key determinant of its competitive advantage.

H10. Product innovation performance will positively affect competitive advantage.

### Research model

**Fig 2:**
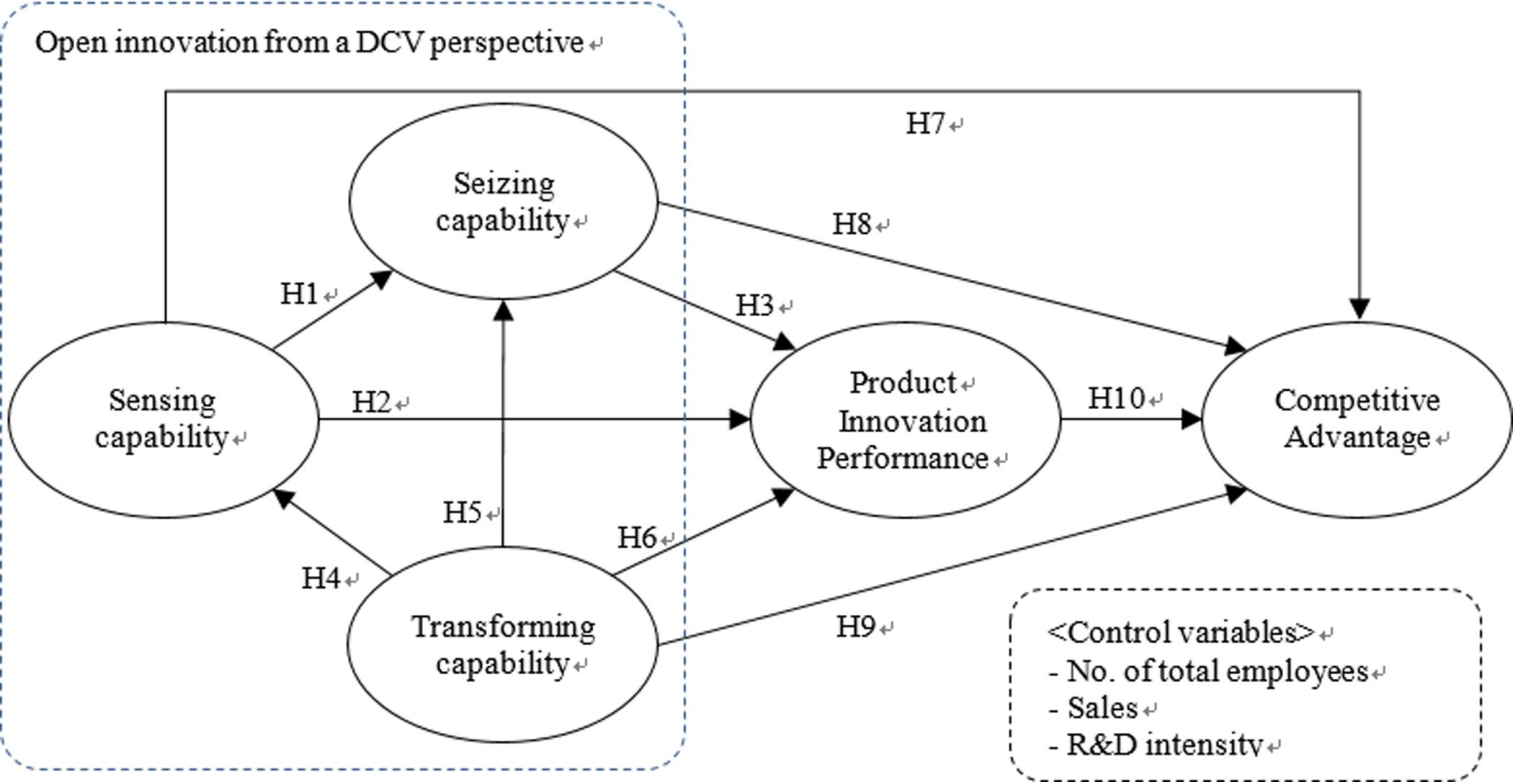
Proposed search model.

Fig 2 illustrates the study’s research model. The research model places the chain of open innovation → product innovation performance → competitive advantage at the central. For the purpose of reviewing the relationship among the three components of dynamic capability required for open innovation, this study examines the relationship between sensing capability, seizing capability and transforming capability as presented in the previous hypothesis. Lastly, control variables including the number of total employees, sales per the number of total employees, and R&D intensity per the number of total employees are employed to limit the potential for bias from confounding effects.

## Methodology

### Data and analysis method

Data was collected from the 2014 Korean Innovation Survey (KIS) to validate the proposed research model. The KIS is a nationwide survey, recurring every 2 to 3 years, which addresses the innovation activities and financial results for all registered firms. The questions in the KIS are based on the Oslo Manual, the third edition [31]. The KIS is comprehensive as the survey includes direct measures of innovation and financial performance along with a wide variety of factors that influence innovation.

The study's target population is Korean manufacturing companies established before 2011 with over 10 employees. 46,101 companies satisfied these criteria in the KIS Database. 4,031 firms which met the study criteria were selected for analysis using a stratified sampling. Firms that reported no innovation activities within the past three years were excluded from the sampling in addition to responses with missing or erroneous data, resulting in a final sample of 987 manufacturers. The firms included in the final sampling are categorized into 23 industries (see Table 1).

**Table 1:** Samples of this study.

KSIC*	Industry (Manufacturing sector)	A population		Selected samples	
10	Food	2,524	5.5%	59	6.0%
11	Beverages	151	0.3%	6	0.6%
13	Textile	2,287	5.0%	19	1.9%
14	Wearing apparel, fur	1,236	2.7%	10	1.0%
15	Leather, shoes	436	0.9%	4	0.4%
16	Wood	581	1.3%	4	0.4%
17	Pulp, paper	1,159	2.5%	13	1.3%
18	Printing, paper press	913	2.0%	7	0.7%
19	Coke, petroleum refining	114	0.2%	3	0.3%
20	Chemical compounds	1,893	4.1%	80	8.1%
21	Medicine and medical supplies	352	0.8%	33	3.3%
22	Plastic, rubber	3,985	8.6%	66	6.7%
23	Non-metallic minerals	1,791	3.9%	29	2.9%
24	Basic metals	2,090	4.5%	19	1.9%
25	Fabricated metals	6,356	13.8%	99	10.0%
26	Electronic components, telecommunication	3,196	6.9%	149	15.1%
27	Medical, precision machinery	1,629	3.5%	54	5.5%
28	Electrical machines	3,023	6.6%	91	9.2%
29	Machinery	6,924	15.0%	136	13.8%
30	Automobiles	2,893	6.3%	69	7.0%
31	Transportation equipment	973	2.1%	11	1.1%
32	Furniture	836	1.8%	15	1.5%
33	Others	759	1.6%	11	1.1%
**Total**		46,101	100.0%	987	100%

*KSIC: Korea Standard Industry

Partial least squares (PLS) was used to analyse the data sampling. PLS is accepted as an appropriate statistical model for structural path analysis and allows the testing of hypotheses with formative latent variables [32]. PLS is an appropriate method for this study since the research model includes structural paths with formative latent variables (i.e., sensing capability, seizing capability, and transforming capability). The data analysis was performed using Smart PLS (version 2.0.M3).

The research model includes reflective as well as formative constructs. The reflective measurement theory is based on the assumption that latent constructs cause the measured variables and measurement error results in an inability to fully explain these measures. The formative measurement theory is based on the assumption that the measured variables cause the construct and measurement errors are the inability to fully explain the construct [32]. Reflective items are representative of the same conceptual domain while formative items define the construct. Sensing capability, seizing capability and transforming capability are formative constructs in the research model while product innovation performance and competitive advantage are reflective constructs.

#### Dynamic capability (DC)

DC was decomposed into sensing capability, seizing capability and transforming capability in accordance with Teece (2007) [18]. The seven dimensions of sensing capability were utilized to construct a seven-item formative scale based on previous research [1, 13]. Survey respondents were asked if external knowledge sourcing is used for any innovation activities within the past three years. If the survey respondent indicated that innovation activities were used, the respondent was asked to evaluate the importance of the activities. Each item was rated on a four-point scale ranging from ‘0’ (“none”) to ‘3’ (“strongly agree”).

Seizing capability was measured using a five-item formative scale. Participants were asked whether their firm introduces/implements various innovations in following areas: collaborative R&D, external R&D contract, machine or software acquisition, external knowledge acquisition, new design [31, 33]. Each item is a binary variable coded ‘1’ if the firm introduces/implements the activity and ‘0’ otherwise.

Collaborative R&D refers to R&D activities conducted in collaboration between an internal organisation and other organisation based on a contract. An external R&D contract indicates R&D activities conducted by a sub-contracted organisation based on an outsourcing contract. Machine or software acquisition means the obtaining of a new machine, equipment or software to be utilized for a new or significantly enhanced product or process. External knowledge acquisition means the purchase of knowhow, intellectual property right or invention that was possessed by other organisation. New design refers to internal or outsourced activities to devise or revise the shape or appearance of product.

Transforming capability was measured using a four-item formative scale. Participants were asked whether their firm introduces/implements organisational innovation in following areas: business practices, workplace organisation, external relations, and product placement [31, 34]. Each item is a binary variable coded ‘1’ if the firm introduces/implements the activity and ‘0’ otherwise.

Business practices include initiating new methods for organizing routines and procedures to conduct work, such as supply chain management, knowledge management, business process re-engineering, quality management, and education/training. Workplace organisation include initiating new methods for delegating responsibility and decision making among employees, but also includes the integration of new business activities. External relations involve fostering new ways of organizing relations with external organisations. Examples of external relations include establishing new collaborations with research organisations or customers, new methods of integration with suppliers, as well as outsourcing organisational activities. Product placement includes both the channels that firms select to sell their products and also how those channels are designed to best market their products.

#### Product innovation performance (PIP)

Prajogo and Ahmed (2006) designed a construct for measuring PIP based on criteria that was conceptualized in previous innovation studies [23] (e.g., [35]). The PIP criteria include the level of novelty of new products, the speed of new product development, the number of new products introduced to the market, and the number of new products that are first-to-market.

In this study, PIP was measured using a four-item reflective scale. Survey respondents were asked about the degree of various outcomes for the variety of products, replacement of old products, early market entrants, and quality enhancement of products. Survey respondents rated all items on a four-point scale ranging from ‘0’ (“none”) to ‘3’ (“strongly agree”).

#### Competitive advantage (CA)

CA could be captured with marketable outputs of innovative products like revenue, ratio of new product sales, and new product success rate [36]. Link and Scott (2010) operationalize CA as innovative sales productivity, which is the ratio of sales attributed to new products divided by the total number of employees [37]. CA is measured in this study using innovative products’ sales ratio and innovative products’ sales per employee. Table 2 summarizes the measurements employed in this study along with relevant studies that support the use of these measurements.

**Table 2:** Variables and measurements.

Constructs	Indicators	References
Sensing Capability	Suppliers, Customers (public sector), Customers (private sector), Competitors (or other firms), Private services (consulting), Universities, Public/Private R&D institutions	[1],[13]
Seizing Capability	Collaborative R&D, External R&D contract, Machine or Software acquisition, External knowledge acquisition, New Design	[31],[33]
Transforming Capability	Business practices, Workplace organisation, External relations, Product placement	[31],[34]
Product Innovation Performance	Variety of products, Replacement of old products, Early market entrants, Quality enhancement of products	[23],[35]
Competitive Advantage	Innovative products’ sales ratio, Innovative products’ sales per employee	[36],[37]

## Results

### Measurement model

A confirmatory factor analysis (CFA) was conducted to test the measurement model. The convergent and discriminant validity of the constructs were examined to validate the measures employed. The composite reliability (CR) for each scale was calculated to analyse the internal consistency of the latent variables [38, 39]. Reliability coefficients of 0.70 or higher are generally considered adequate [40]. The CR values of all reflective constructs were above 0.70 (see Table 3).

**Table 3:** Factor loadings and AVE of latent variables.

Constructs	Indicators	Loadings	AVE	Composite Reliability
Sensing Capability	Suppliers Customers (public) Customers (private) Competitors (or other firms) Private services (consulting) Universities Public/Private R&D institutions	na	na	na
Seizing Capability	Collaborative R&D External R&D contract Machine or Software acquisition External Knowledge acquisition New Design	na	na	na
Transforming Capability	Business practices Workplace organisation External relations Product placement	na	na	na
Product Innovation Performance	Variety of products Replacement of old products Early market entrants Quality enhancement of products	0.694*** 0.764*** 0.806*** 0.657***	0.537	0.821
Competitive Advantage	Innovative products’ sales ratio Innovative products’ sales per employee	0.834*** 0.987***	0.835	0.910

na: Loading, AVE and Composite Reliability are not applicable to formative constructs

***p < .01

Convergent validity is assessed by examining both factor loadings and the average variance extracted (AVE). The factor loading for each latent construct item was significant at the 0.01 level (see Table 3). AVE measures the overall proportion of variance accounted for in each latent construct item. Convergent validity was exhibited for each latent construct item as all shared variances were well above the recommended threshold level of 50% [40] (see Table 3). Discriminant validity was exhibited for each measure using item loadings, cross-loadings, the square root of the AVE, and a correlation matrix (see Tables 4 and 5). The CFA results support the reliability and validity of each measure. Additionally, we conducted endogeneity test for three latent constructs including seizing capability, product innovation performance, and transforming capability. We adopted a verification technique using instrumental variable proposed in prior studies [41–44] As a result, there is no significant endogeneity problems (see Appendix).

**Table 4:** Discriminant validity (cross-loadings).

Indicators	Sensing Capability	Seizing Capability	Transforming Capability	Product Innovation Performance	Competitive Advantage
Suppliers	**0.507**	0.219	0.214	0.176	-0.014
Customers (public)	**0.353**	0.166	0.130	0.272	-0.044
Customers (private)	**0.397**	0.211	0.113	0.133	0.072
Competitors (or other firms)	**0.519**	0.247	0.187	0.219	0.056
Private services (consulting)	**0.608**	0.285	0.226	0.106	0.042
Universities	**0.748**	0.363	0.261	0.183	-0.010
Public/private R&D institutions	**0.641**	0.340	0.183	0.136	0.029
Collaborative R&D	0.434	**0.706**	0.346	0.140	0.004
External R&D contract	0.325	**0.576**	0.290	0.133	0.045
Machine or software acquisition	0.197	**0.546**	0.364	0.137	-0.052
External knowledge acquisition	0.248	**0.427**	0.223	0.078	0.053
New design	0.282	**0.722**	0.380	0.291	0.049
Business practices	0.275	0.417	**0.757**	0.222	-0.038
Workplace organisation	0.262	0.440	**0.768**	0.212	-0.084
External relations	0.301	0.450	**0.839**	0.273	0.003
Product placement	0.209	0.284	**0.623**	0.298	-0.003
Variety of products	0.179	0.206	0.190	**0.694**	-0.019
Replacement of old products	0.221	0.170	0.241	**0.764**	-0.017
Early market entrants	0.246	0.257	0.304	**0.806**	0.050
Quality enhancement of products	0.190	0.201	0.226	**0.657**	0.080
Innovative products’ sales ratio	0.098	0.162	0.063	0.136	**0.834**
Innovative products’ sales per employee	-0.008	-0.012	-0.061	0.004	**0.987**

**Table 5:** Correlations of latent variables.

Construct	Sensing Capability	Seizing Capability	Transforming Capability	Product Innovation Performance	Competitive Advantage
Sensing Capability	na				
Seizing Capability	0.483	na			
Transforming Capability	0.352	0.534	na		
Product Innovation Performance	0.289	0.288	0.334	*0.733*	
Competitive Advantage	0.017	0.029	-0.035	0.036	*0.914*

Diagonal elements in italic font style are the square root of the AVE

na: AVE is not applicable to formative constructs

### Hypotheses testing

**Fig 3:**
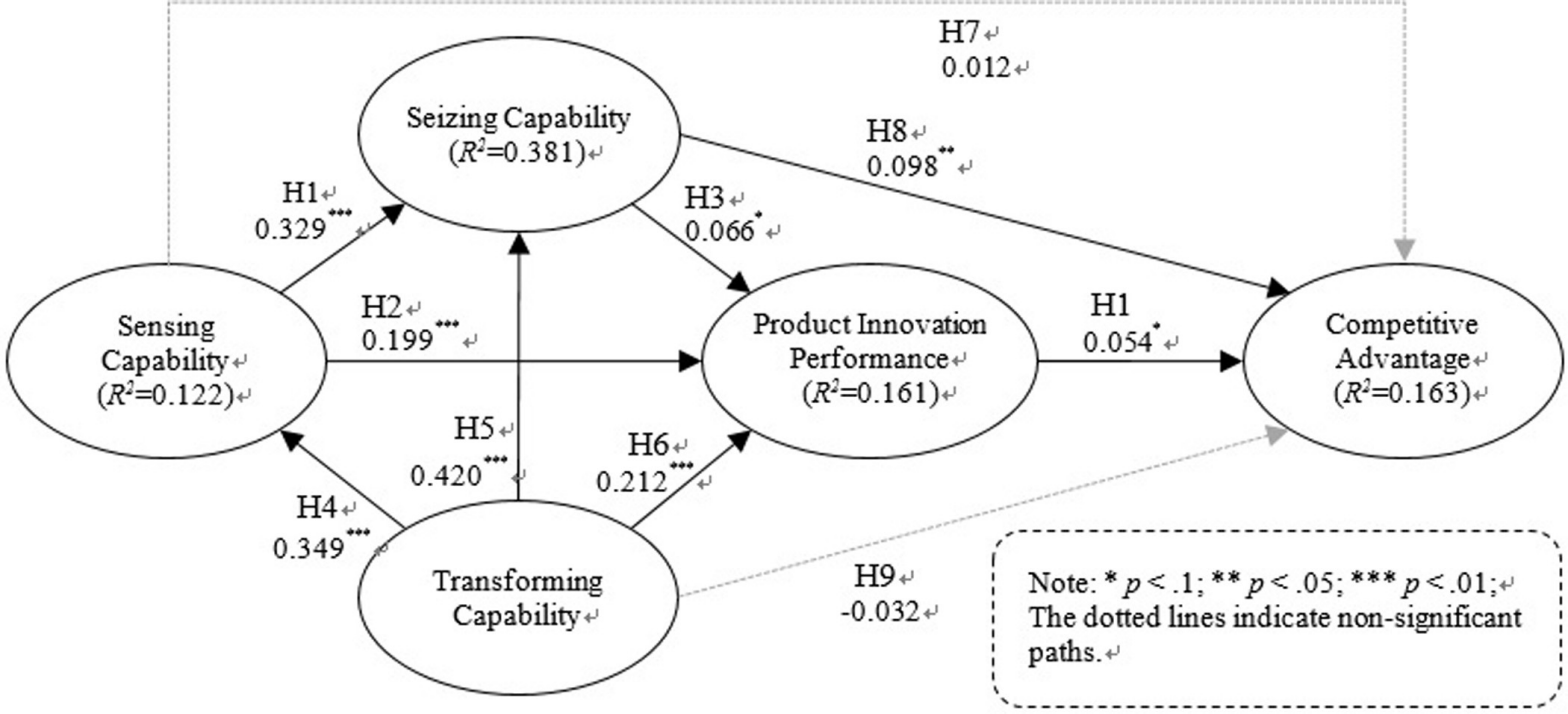
Hypotheses tests results.

The structural equation modelling results are presented in Fig 3 and the hypothesis tests are summarized in Table 6. Eight out of the ten hypotheses were supported in the data analysis. A bootstrapping re-sampling technique was employed to calculate the corresponding t-values for each hypothesized relationship.

**Table 6:** Summary of hypotheses testing results.

Hypothesis		Path coefficient	t-value	Outcome
H1:	Sensing ca. → Seizing ca.	0.329***	9.707	Supported
H2:	Sensing ca. → PIP	0.199***	5.914	Supported
H3:	Seizing ca. → PIP	0.066*	1.829	Supported
H4:	Transf. ca. → Sensing ca.	0.349***	11.758	Supported
H5:	Transf. ca. → Seizing ca.	0.420***	13.630	Supported
H6:	Transf. ca. → PIP	0.212***	5.917	Supported
H7:	Sensing ca. → CA	0.012	0.328	Rejected
H8	Seizing ca. → CA	0.098**	2.394	Supported
H9	Transf. ca. → CA	-0.032	1.035	Rejected
H10	PIP → CA	0.054*	1.814	Supported

*p < .1,

**p < .05,

***p < .01

The data analysis results support H1, indicating that sensing capability positively affects seizing capability. The relationship between transforming capability and sensing capability was significant, which supports H4, and the relationship between transforming capability and seizing capability also significant, which supports H5.

The relationship between the three components of DC—sensing capability, seizing capability and transforming capability and PIP is statistically significant. Therefore, the data analysis results support H2, H3, and H6. When considered the relationship between DC and CA, H8 is supported, which describes the relationship between seizing capability and competitive advantage. However, the direct relationships between sensing capability and CA, and between transforming capability and CA, are not statistically significant. Thus, H7 and H9 are not supported.

Lastly, PIP positively influences CA. Thus, the data analysis results support H10.

## Discussions

By adopting DCV approach, this study provides empirical evidence of the chain effect from firm’s open innovation activities (dynamic capability) through product innovation performance (PIP) to competitive advantage (CA). This chain effect confirms that company’s strengthening of dynamic capability (DC) positively influences CA acquisition, having a positive effect on the market performance of innovative products.

The previous studies are able to explain only some of the individual relationships between DC, PIP and CA while the relationship among these constructs within one integrated framework has not been presented [26, 28]. This study shows through empirical analyses the direct and/or indirect relationship that the higher a company’s DC is, the more CA it has. These results indicate that manufacturers' efforts in the rapidly changing environment to increase adaptability and strategic flexibility by strengthening DC may lead to product innovation opportunities and furthermore sustainability in the market [45].

This study extends the theoretical discussion of DCV by revealing the relationships between the three components of DC. Unlike previous studies, this study tried to measure on the basis of the specific characteristics of sensing capability, seizing capability and transforming capability. This allows this study to get a closer look at the impact of the DC components on CA and reduce the halo effect where the aggregate properties define individual properties [46].

A business must constantly explore technology and the market to recognize and create opportunities. To do so, the organisation's capability to support it must also be improved [14]. This study confirms that transforming capability is a prerequisite affecting both sensing capability and seizing capability. Transforming capability enables continuous growth by reassembling and reconfiguring the resources and organisational structures of companies in a constantly changing industrial environment [18]. Companies require new technologies and capabilities in a volatile and changing environment, so they must procure them from the outside and reorganize them in harmony with the existing capabilities.

Previous theories have contradicted ideas on external sensing activities and those supporting activities related to transforming capability [21]. Placing personnel in positions for external collaboration, creating a dedicated organisation, building a knowledge management system or communicating and collaborating with external channels incur expenses. Investment in activities involved in external knowledge sourcing and support for those activities accompany risks. The result of this study shows, however, that transforming capability is an important factor (β = 0.349) leading to successful sensing capability. Furthermore, transforming capability has a direct positive effect on product innovation performance (β = 0.212). Thus, transforming capability, which builds a foundation for the absorption, distribution, sharing and utilization of knowledge for the collaboration with various external partners, is an important prerequisite of sensing capability.

The direct effect of sensing capability and transforming capability on CA is not statistically significant; however, both cases show indirect effects that have positive impact on the PIP and, in a sequential manner, CA. Further analysis of the total effect size (β = 0.254) showed that seizing capability (β = 0.102) had the greatest effect on CA and was followed by sensing capability (β = 0.056), PIP (β = 0.054) and transforming capability (β = 0.042).

Since the direct effect of transforming capability on CA is not statistically significant, the coexistence of its positive and negative effects can be considered. This study considered the aspect of enhancing market performance by updating sales business division, marketing and sales organisation, and enhancing market share or expanding sales area through relationship changes such as strategic collaboration with other companies. However, due to the negative impacts of transforming capability on CA, for example change costs, learning costs, switching costs to a new organisational system, cultural conflicts, complaints of members or work inefficiency until a new system becomes accustomed [45], it can be inferred that the positive influence was offset. In other words, if organisational innovation has been carried out effectively, market performance will appear; however, it can be considered to be in line with Damanpour and Evan (1984) that adverse effects may occur because it may accompany confusion and inefficiency [47].

The study confirms that sensing capability has a positive effect on seizing capability, PIP and finally the improvement of CA rather than affects in itself competitive advantage. As the opportunities for product innovation vary depending on the subject of cognitive activity, companies need to pursue product innovation strategies for not just short-term growth but mid- to long-term growth by broadening the scope of cognitive activities.

The depth and breadth of knowledge can vary depending on whether sensing aims at explorative activities for new product development or for exploitive activities for improving existing products [12]. It is possible, therefore, to minimize the transaction costs incurred by external collaboration when investing the expenses with clear goals after determining the best possible partners for cooperation. It is generally known that knowledge required for product improvement is likely to be provided by internal experts but knowledge necessary for new product development is highly likely to be introduced from users or academic communities outside the company [48].

Focusing on activities that increase PIP is more effective than linking transforming capability to direct product sales. Companies need to operate TFT or place qualified personnel in right positions to boost the effectiveness of internal R&D activities and perform continuous education and training to promote product innovation performance. In addition, care must be taken to minimize the adverse effects of these activities. The spread of innovation causes resistance because it requires change to users [49]. For this reason, the introduction of measures to overcome innovation resistance before acceptance and diffusion of innovation should also be considered [50].

Knowledge acquired from the outside includes not only technical aspects but also ideas about non-technical aspects such as price, distribution and promotion. Considering that economic conditions, purchase accessibility and consumer awareness as well as superiority in performance are important factors for product diffusion, discovering knowledge that has direct impact on it is a crucial factor to determine market performance. Therefore, it is important not only to focus on finding technical elements in developing products based on external collaboration but also to determine the type of knowledge for non-technical innovation and to obtain the necessary knowledge from the most appropriate sources. Furthermore, the effect of acquired knowledge can be maximized when it is organically shared with the marketing department and executing organisations via the organisation’s system [14].

## Conclusions

The performance of an organisation is determined by whether it has core assets and how effectively the assets can be utilized by the organisation. In particular, in a rapidly changing environment, the ability to sense and seize intangible assets created by internal and external members, and transforming capability to transform existing knowledge into resources that are used to respond to environmental change is the key sources contributing to the creation and maintenance of competitive advantage.

The results of this study verify that the three dynamic capabilities would be helpful for companies to carry out open innovation and that although individual capabilities have unique features, respectively, they are integrated rather than acting separately and affect product innovation performance and competitive advantage in a comprehensive manner. The significance of this study can be found that it also moved one step further from previous studies that focused on explaining individual relationships in order to develop and verify a research model within one unified theoretical framework between dynamic capability and competitive advantage.

However, this study has limitation in that the data from Korean manufacturing companies with experience in innovations were used for analysis, so it is important to be careful in interpreting and generalizing the results. The exclusion of certain groups from the sample can cause problems with selection bias. Nevertheless, researchers have carried out a variety of empirical studies for the early adopters. This has the advantage of being able to identify factors that significantly affect acceptance of innovations. This study also focuses on identifying the causal relationships among innovation related activities of companies with innovative experience [51]. Therefore, it is worth noting that the results are only significant for groups with experience in innovation.

Future research needs to be focused on the following two priorities. First, for this study, Korea’s manufacturing industry data (KIS 2014) were used for the analysis. It is necessary to perform additional researches on how these results will appear in other industries. Each industry has different subjects and fields of innovation that matter. However, regardless of the type of industry, it is clear that knowledge and skills are important inputs to determine the productivity and innovation of the final product. It will be an interesting research topic to examine how the relationship between dynamic capability and competitive advantage in service industries. Unlike that have different characteristics of innovation from manufacturing industry, innovations in service sector occur in processes and business models through frequent knowledge exchange from suppliers and customers.

Second, open innovation can be divided into three dimensions—inbound, outbound and coupled activities. The scope of this study is limited to inbound open innovation. Inbound open innovation indicates the acquisition and internalization of external technology or knowledge, while outbound open innovation refers to the external exploitation of internal knowledge using open innovation strategies such as outward intellectual property licensing. Thus, outbound open innovation is expected to relatively focus on sensing and seizing capabilities for business opportunities and partners for collaboration rather than transforming capabilities. Research on these issues will expand our understanding on the relationship between open innovation and firm’s dynamic capability and its consequences on business performance.

## Supporting information

S1 Fig Analysis of endogeneity. We applied the instrumental variable (IV) approach and tested using the WarpPLS 6.0 program based on several recent studies [4, 22, 41] dealing with endogeneity in PLS-SEM problems. The relationship between open innovation and company’s competitive advantage, and organisational capabilities.
(TIF)

S1 Dataset (XLSX)
