# Supplementary figures and images for: How does open innovation lead competitive advantage? A dynamic capability view perspective
Source: PLoS One. 2019 Nov 20;14(11):e0223405. doi: 10.1371/journal.pone.0223405 (PMC6867631; doi:10.1371/journal.pone.0223405)

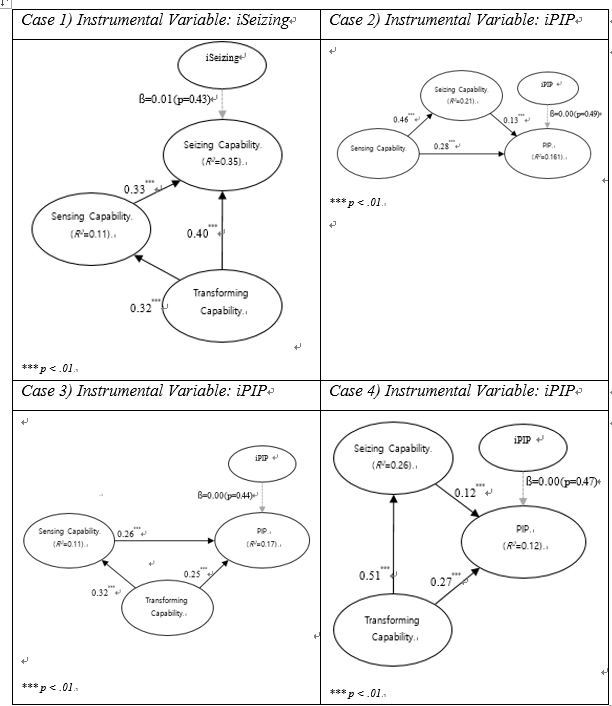

Supplement: S1 Fig — We applied the instrumental variable (IV) approach and tested using the WarpPLS 6.0 program based on several recent studies [4, 22, 41] dealing with endogeneity in PLS-SEM problems. The relationship between open innovation and company’s competitive advantage, and organisational capabilities. (TIF) [file pone.0223405.s001.tif]
